# Topoisomerase inhibitor amonafide enhances defense responses to promote longevity in *C. elegans*

**DOI:** 10.1007/s11357-025-01599-5

**Published:** 2025-03-14

**Authors:** Iman Man Hu, Ana Serna, Stacia Everts, Lale Güngördü, Bauke V. Schomakers, Ellen A. A. Nollen, Arwen W. Gao, Riekelt H. Houtkooper, Georges E. Janssens

**Affiliations:** 1 Laboratory Genetic Metabolic Diseases, Amsterdam UMC Location University of Amsterdam, Meibergdreef 9, Amsterdam, The Netherlands; 2 Amsterdam Gastroenterology Endocrinology and Metabolism Institute, Amsterdam, The Netherlands; 3 Amsterdam Cardiovascular Sciences Institute, Amsterdam, The Netherlands; 4 European Research Institute for the Biology of Ageing, University Medical Center Groningen, University of Groningen, Groningen, The Netherlands; 5 Core Facility Metabolomics, Amsterdam UMC Location University of Amsterdam, Meibergdreef 9, Amsterdam, The Netherlands

**Keywords:** *Caenorhabditis elegans*, Longevity, Topoisomerase inhibitors

## Abstract

Aging is a major risk factor for disease, and developing effective pharmaceutical interventions to improve healthspan and promote longevity has become a high priority for society. One of the molecular pathways related to longevity in various model organisms revolves around lowering AKT1 levels. This prompted our in silico drug screen for small molecules capable of mimicking the transcriptional effects of *AKT1* knockdown. We found topoisomerase inhibitors as a top candidate longevity-drug class. Evaluating multiple compounds from this class in *C. elegans* revealed that the topoisomerase inhibitor amonafide has the greatest benefit on healthspan and lifespan. Intriguingly, the longevity effect of amonafide was not solely dependent on *DAF-16/FOXO*, the canonical pathway for lifespan extension via *AKT1* inhibition. We performed RNA-seq on amonafide-treated worms and revealed a more youthful transcriptional signature, including the activation of diverse molecular and cellular defense pathways. We found the mitochondrial unfolded protein response (UPR^mt^) regulator *afts-1* to be crucial for both improved healthspan and extended lifespan upon amonafide treatment. Moreover, healthspan was partially dependent on the immune response transcription factor *zip-2* and the integrated stress response transcription factor *atf-4*. We further examined the potential of amonafide in age-related disease. Treating a *C. elegans* model for Parkinson’s disease with amonafide improved mobility. In conclusion, we identified amonafide as a novel geroprotector, which activates mitochondrial-, pathogen-, and xenobiotic-associated defense responses that—though more studies are needed—may serve as a candidate for Parkinson’s disease therapy.

**Supplementary Information:**

The online version contains supplementary material available at 10.1007/s11357-025-01599-5.

## Introduction

Longevity is a multifaceted trait influenced by many factors, encompassing genetics, lifestyle choices, environmental conditions, and healthcare practices. These factors also shape the health trajectory and age-related decline of populations [1]. Developing effective interventions to reduce the incidence of late-life disorders and improve lifespan has become a high priority for scientific research and society. One approach to developing such interventions involves the use of small-molecule drugs due to its adaptability, control, and convenience of application [2]. As our understanding of longevity mechanisms advances, an increasing number of geroprotective molecules—compounds that are aimed at preserving the “gerontological” phase of life—are being identified and studied [2, 3]. Among the most promising strategies for developing geroprotective compounds is targeting key components in the nutrient-sensing network [4].

The insulin/insulin-like growth factor-1 (IGF-1) signaling (IIS) pathway constitutes a conserved nutrient-sensing system that coordinates growth, differentiation, and metabolism in response to changing environmental conditions and nutrient availability [5]. Evidence from various model organisms underscores that the IIS pathway is an evolutionarily conserved mechanism influencing longevity [6, 7]. Indeed, genes within the IIS pathway were among the initial set demonstrated to extend the lifespan of *Caenorhabditis elegans* (*C. elegans*) [8, 9]. The canonical IIS pathway in *C. elegans* comprises insulin-like ligands, the insulin/IGF-1 receptor tyrosine kinase DAF-2, and downstream signaling components such as AGE-1 (phosphatidylinositol-3-kinase), PDK-1 (phosphoinositide-dependent protein kinase-1), and the serine/threonine kinases AKT-1/AKT-2, and the primary downstream effector DAF-16 [10]. Under conditions of decreased IIS, downstream transcription factors translocate from the cytoplasm to the nucleus, inducing changes in gene expression, ultimately leading to lifespan extension [11, 12].

Recapitulating the longevity induced by IIS inhibition has been extensively studied with the goal of developing conserved genetic longevity interventions [13]. DAF-16, a major downstream transcription factor of IIS, plays a central role in regulating lifespan, stress resistance, and metabolism [14–17]. Current antiaging approaches targeting DAF-16/FOXO include strategies to enhance its activity through small molecules or by modulating upstream IIS signaling. In a previous study, we outlined a drug screening method that used computational analysis to screen geroprotective compounds mimicking the transcriptome effect of the overexpression of *daf-16/*FOXO [18]. By analyzing transcriptomic signatures that mimic the effects of *daf-16*/FOXO overexpression, this approach enabled the identification of potential small molecules promoting both lifespan and healthspan [18, 19], adding to a growing list of small molecules promoting longevity through *daf-16/*FOXO signaling [20]. The IIS pathway regulates longevity through its downstream effector *daf-16*/FOXO, whose activity is tightly controlled by upstream components, including AKT-1. AKT-1, an essential serine/threonine kinase in the IIS pathway, phosphorylates DAF-16, inhibiting its nuclear localization and activity [10]. In *C. elegans*, inhibition of AKT-1 has been shown to extend lifespan by allowing DAF-16 to enter the nucleus and activate longevity-associated genes [21]. AKT-1 functions as an important regulator of the aging process, influencing key cellular mechanisms related to longevity [22]. Furthermore, haploinsufficiency of *AKT1* leads to lifespan extension in mice [22, 23]. Hence, our hypothesis centers on the potential of screening compounds with transcriptome signatures resembling AKT-1 inhibition. By identifying compounds mimicking the transcriptional effects of AKT-1 inhibition, we aim to modulate key pathways associated with longevity, ultimately extending healthspan and lifespan.

In this study, we employed a transcriptomics-based in silico drug screening to identify compounds with a transcriptional signature similar to AKT-1 knockdown. Topoisomerase inhibitors emerged from this screen as a distinct category of potential longevity enhancers. We evaluated the impact of three types of topoisomerase inhibitors in *C. elegans* on healthspan (as measured by mobility) and lifespan. All tested topoisomerase inhibitors demonstrated significant improvement of mobility during aging. The topoisomerase inhibitor amonafide, in particular, had a marked impact in enhancing healthspan and extending lifespan. The observed improvements induced by amonafide were not simply dependent on *daf-16*. Through subsequent RNA-seq analysis and fluorescent reporter measurements, we found that amonafide activated a diverse range of defense responses. While a substantial subset of these activated defense responses was essential for the observed enhancements in healthspan, only the *afts-1*-mediated mitochondrial unfolded protein response (UPR^mt^) was required for both the improved healthspan and the extended lifespan following amonafide treatment. We further tested the geroprotective effect of amonafide in α-Syn (UM10), a worm model of Parkinson’s disease (PD), which revealed that amonafide reduces disease symptoms in PD worms. In summary, our research identifies topoisomerase inhibitors as a potential new class of geroprotectors, with amonafide as a novel protective agent, activating mitochondria-, pathogen-, and xenobiotic-associated defenses responses to promote healthspan and lifespan.

## Results

### Topoisomerase inhibitors demonstrate geroprotective effects

**Fig. 1:**
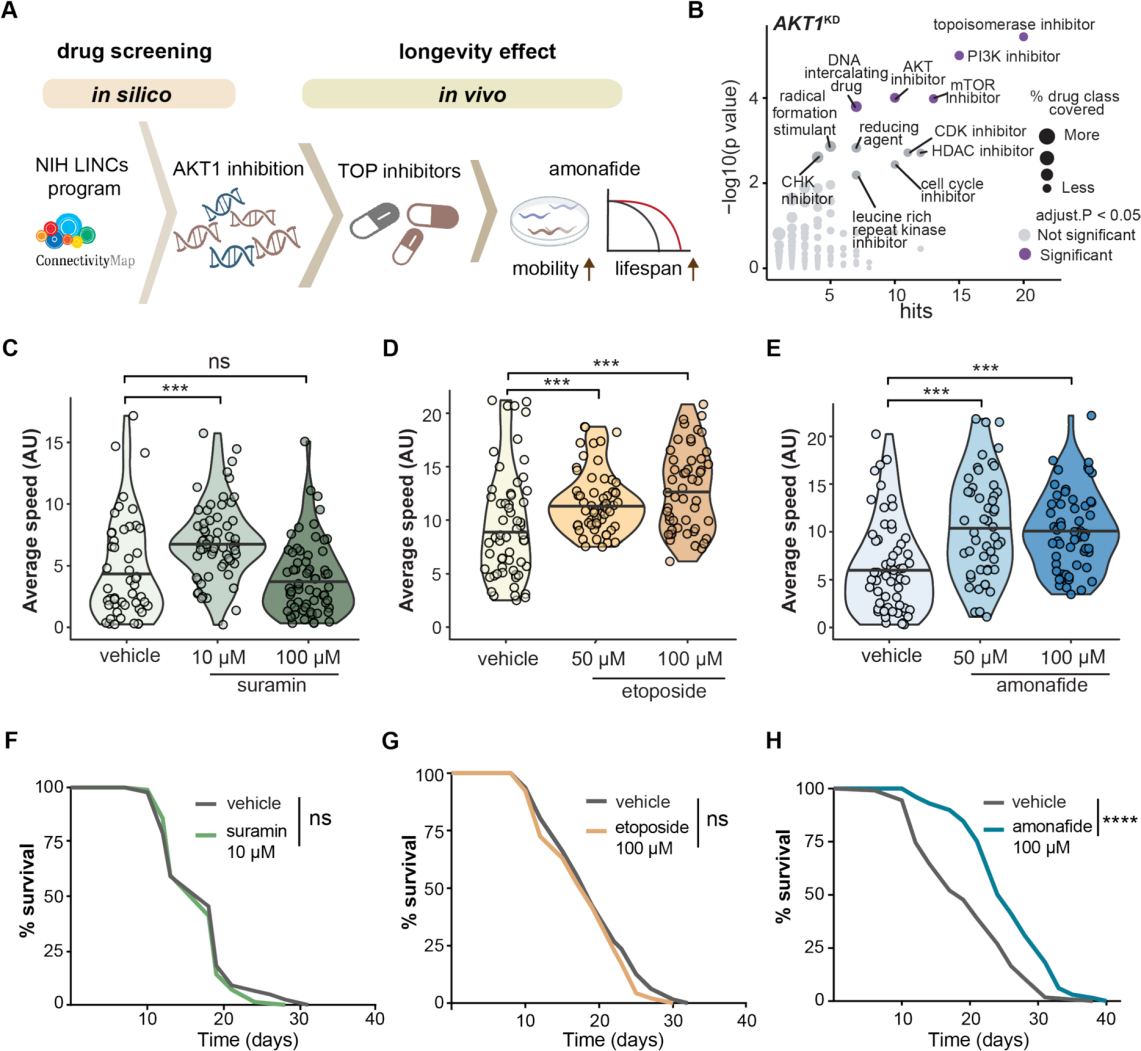
Topoisomerase inhibitors exhibit geroprotective effects in *C. elegans*. **A** Diagram shows how this study identified amonafide as a geroprotective compound, using in silico and in vivo methods. **B** Results of drug classes enriched as mimicking the transcriptional signature of *AKT1* knockdown in cells. Drug classes are plotted against the number of hits (*X*-axis) and the *p*-value significance of enrichment (*Y*-axis, with an adjusted *p*-value threshold of < 0.05 for inclusion). The size of each dot correlates with the percentage of hits within that drug class. **C** Violin plot representing the mobility of *C. elegans* (N2) under vehicle (water) and suramin treatment. **D** Violin plot representing the mobility of *C. elegans* (N2) under the vehicle (DMSO) and etoposide treatment. **E** Violin plot representing the mobility of *C. elegans* (N2) under the vehicle (DMSO) and amonafide treatment. **C**–**E** *Y*-axis shows the average moving speed of *C. elegans* at day 13. The bar in the center of the violin plot represents the median value of mobility. The statistical analysis was performed using a one-way ANOVA test followed by Tukey post hoc test; groups were compared to vehicle. *** represents *p*-value < 0.001, and “ns” represents not significant. **F** Lifespan curves of *C. elegans* (N2) treated with vehicle (water) or suramin. **G** Lifespan curves of *C. elegans* (N2) treated with vehicle (DMSO) or etoposide. **H** Lifespan curves of *C. elegans* (N2) treated with vehicle (DMSO) or amonafide. **F**–**H** *p*-values represent a comparison with the controls calculated using a log-rank test. **** represents *p*-value < 0.0001, and ns represents not significant

In order to identify small molecules capable of mimicking the transcriptional signature of *AKT1* knockdown, we designed an in silico and in vivo drug discovery pipeline, ultimately resulting in the identification of amonafide as a novel geroprotector (Fig. 1A). The approach consisted of three main sections: (a) drug screening based on public databases and computational analysis, (b) assessment of candidates for geroprotective effects in *C. elegans*, and (c) validation of the longevity mechanism of the lead candidate, amonafide (Fig. 1A). Using the *AKT1* knockdown transcriptional signature available in the Library of Integrated Network-based Cellular Signatures (LINCS) database [24, 25], we used the same drug screening method as previously used by our lab [18, 19] to identify compounds that induce effects akin to *AKT1* knockdown. A ranked list of small molecules possessing transcriptomics signatures most similar to the transcriptomic signature of *AKT1* inhibition across various cell strains was generated using the publicly available data and user interface of the LINCS database [24, 25]. We further refined this list by searching for drug classes that were enriched among the highest-ranked small molecules (Fig. 1B). This analysis allowed us to identify prevalent drug classes in our list relative to the entire dataset. Notably, AKT inhibitors, mTOR inhibitors, and PI3 kinase inhibitors all emerged as strongly enriched drug classes, aligning with the *AKT1* inhibition transcriptional signature (Fig. 1B). Given that AKT inhibitors, mTOR inhibitors, and PI3 kinase inhibitors are recognized for their ability to extend lifespan and operate within the PI3K-AKT longevity pathway [26–28], our findings suggest that this compound screening approach effectively identified compounds mimicking AKT1 inhibition. Several AKT1 inhibitors revealed as hits in our screen included A-443644, staurosporine, and hexamethylenebisacetamide. Notably, topoisomerase inhibitors emerged as the most prominent class of compounds exhibiting a transcriptomic profile closely resembling *AKT1* inhibition (Fig. 1B). Topoisomerase inhibitors, typically used as chemotherapeutic agents, interfere with the topoisomerase enzymes (topoisomerase I and II), governing changes in DNA structure [29]. Despite their known chemotherapeutic role, there is currently no established link between topoisomerase inhibitors and longevity regulation, drawing our attention to further investigate this drug class.

We focused our study on three distinct topoisomerase inhibitors: suramin [30], etoposide [31], and amonafide [32]. Suramin, a hexasulfated naphthylurea that inhibits topoisomerase II, has been used in the treatment of trypanosomiasis [30]. Etoposide, which targets topoisomerase II, is a commonly used anti-cancer agent [31]. Amonafide is a DNA intercalating agent that interferes with topoisomerase II activity and is a promising anti-cancer compound [32] (Table S1). Healthspan and lifespan were assessed at two different doses for each topoisomerase inhibitor (Fig. 1C–E). The concentrations of the low doses were set at either 10 μM (suramin) or 50 μM (etoposide and amonafide), relative to the dosing most generally used in cell culture, and the high dose was set at 100 μM for all three compounds. Healthspan was evaluated by measuring the mobility of *C. elegans* treated with either vehicle or each respective topoisomerase inhibitor. Mobility assays conducted on day 13 of adulthood revealed a significant improvement in the average movement of worms at 10 μM for suramin; however, this effect was not present at 100 μM (Fig. 1C). Both the 50 μM and 100 μM of etoposide significantly increased the mobility of worms (Fig. 1D). A similar beneficial effect was observed with amonafide at both 50 μM and 100 μM doses (Fig. 1E). Further lifespan tests for these beneficial doses of each compound revealed that, despite the healthspan increase observed in certain doses of suramin and etoposide, the lifespan curves showed no significant effects for these two topoisomerase inhibitors (Fig. 1F–G). However, we observed a 25% lifespan extension in worms treated with 100 μM amonafide compared to vehicle controls (Fig. 1H). To further investigate the effects of suramin, etoposide, and amonafide on worm development, we measured the body size of treated worms. Our analysis revealed that worms treated with suramin and amonafide displayed a slightly larger body size compared to the control group, whereas worms treated with etoposide showed a body size similar to that of the controls (S1A Fig). Taken together, these results suggest that the tested topoisomerase inhibitors may have geroprotective properties, with all three tested topoisomerase inhibitors enhancing healthspan in *C. elegans*. Furthermore, demonstrates potential in promoting both healthspan and longevity.

### The roles of *top-2*/TOP2 and *daf-16/FOXO* in amonafide’s healthspan and lifespan extension

**Fig. 2:**
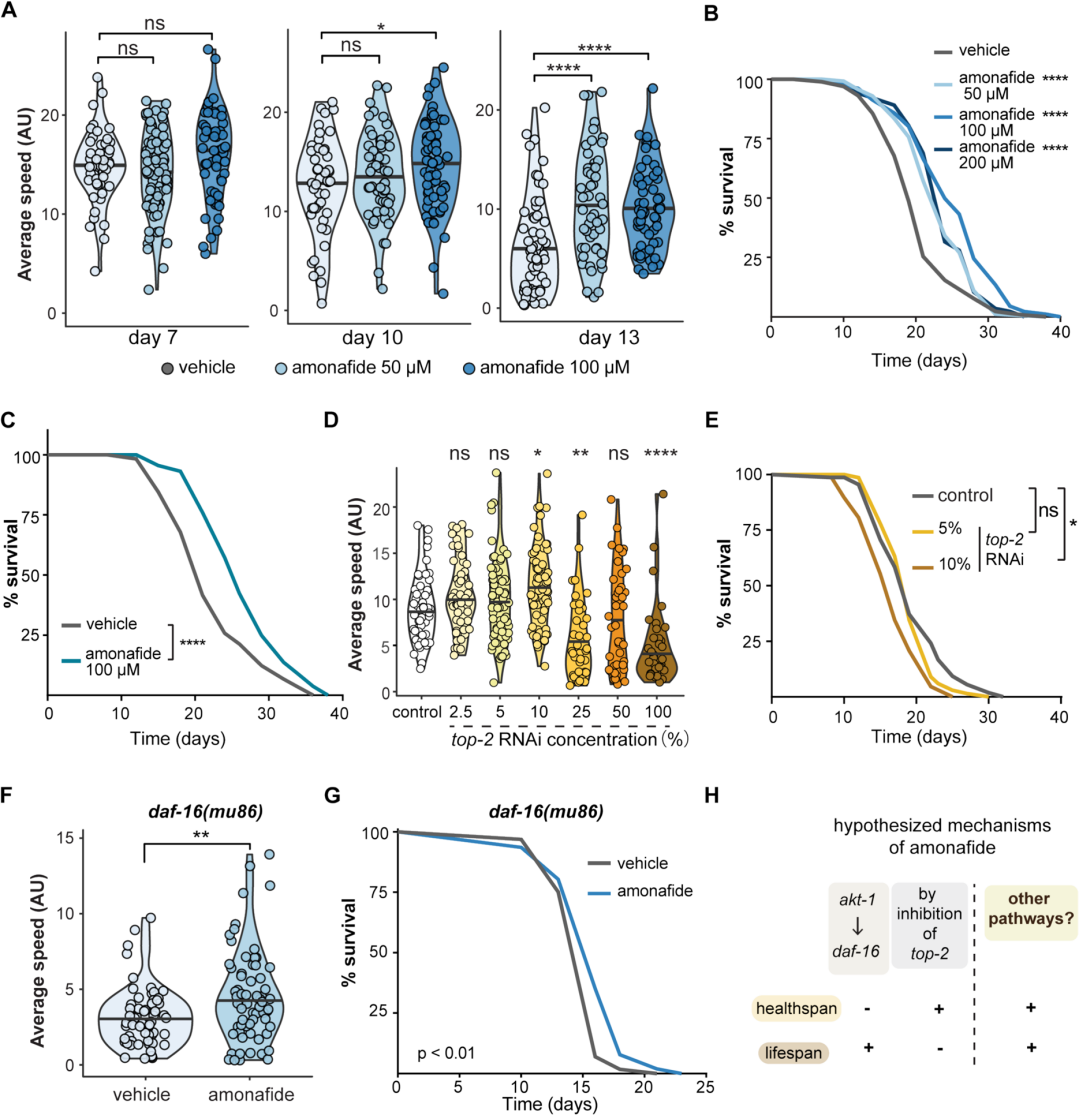
Amonafide improves healthspan and extends lifespan. **A** Violin plot representing the healthspan of *C. elegans* (N2) under vehicle (DMSO) or amonafide treatment in different doses as specified. Mobility of worms was measured at three time points as specified. The statistical analysis was performed using a one-way ANOVA test; groups were compared to vehicle. **** represents *p*-value < 0.0001, * represents *p*-value < 0.05, and ns represents not significant. **B** Lifespan curves of *C. elegans* (N2) treated with vehicle (DMSO) or amonafide in different doses as specified. *p*-values were calculated using the log-rank test for comparisons with the control group. *** represents *p*-value < 0.001. **C** Lifespan curves of *C. elegans* (N2) maintained on dead *E. coli* OP50. Worms were treated with vehicle (DMSO) or 100 μM amonafide as indicated. *p*-values were calculated using the log-rank test for comparisons with the control group. **** represents *p*-value < 0.0001. **D** Violin plot representing the mobility of *C. elegans* (N2) under the same conditions as shown in **C** at day 13. The statistical analysis was performed using a one-way ANOVA test followed by Tukey post hoc test; groups were compared to control (HT115). ** represents *p*-value < 0.01, * represents *p*-value < 0.05, and ns represents not significant. **E** Lifespan curves of *C. elegans* (N2) treated with control (HT115), 5% *top-2* RNAi, and 10% *top-2* RNAi. Percentage represents the concentration of *top-2* RNAi bacteria. *p*-values were calculated using the log-rank test for comparisons with the control group. * represents *p*-value < 0.05, and ns represents not significant. **F** Violin plot representing the mobility of *daf-16(mu86)* under treatment of vehicle or 50 μM amonafide. The statistical analysis was performed using a wilcox.test. ** represents *p*-value < 0.01. **G** Lifespan curves of *daf-16(mu86)* treated with vehicle or 50 μM amonafide. The *p*-value represents the comparison with the controls calculated using the log-rank test. **H** Hypothesis of mechanisms underlying the longevity effect of amonafide

Given the pronounced effect of amonafide on lifespan extension and healthspan improvement, we further investigated its impact on healthspan at various time points (day 7, day 10, and day 13). Mobility assays revealed a consistent increase in mobility at all tested time points with 100-μM amonafide treatment (Fig. 2A). The lower dose of amonafide, 50 μM, did not lead to evident improvement of mobility at earlier time points, but improved mobility at day 13 (Fig. 2A). We also observed a trend indicating that amonafide’s positive impact on mobility became more pronounced as the worms aged (Fig. 2A, S1B Fig). In addition to assessing healthspan, we conducted lifespan measurements on worms subjected to different doses (50, 100, and 200 μM) of amonafide. The lifespan curves demonstrated a remarkable extension in lifespan across all three administered doses compared to the control (Fig. 2B). Among the tested dosages, 100 μM amonafide exhibited the most substantial extension of lifespan, while the lifespan curve for 200 μM amonafide overlapped with that of 50 μM amonafide. This observation suggests that elevating the dosage to 200 μM does not confer additional benefits to lifespan (Fig. 2B). To determine whether the beneficial effects of amonafide were mediated through bacterial metabolism, we tested its impact on worms maintained on bacteria killed using UV cross-linking to eliminate bacterial metabolism. Significant lifespan extension was still observed in worms cultured on inactivated bacteria and treated with amonafide compared to the control (Fig. 2C). This result suggests that the lifespan-extending effects of amonafide are likely attributable to direct interactions with the worms rather than reliance on bacterial metabolism.

Amonafide, a naphthalimide derivative, has been investigated as an anticancer agent. Its inhibitory effect on topoisomerase was identified in the late 1980s [33, 34]. Topoisomerases, comprising two major classes, type I (*TOP1*) and type II (*TOP2*), play a crucial role in altering the topological properties of genetic material [29]. Amonafide functions as a DNA intercalating agent, disrupting the activity of *TOP2* [33]. We considered the possibility that the observed improvement in healthspan and lifespan with amonafide treatment may be linked to its inhibition of *TOP2*. To verify this connection, we measured the healthspan of *C. elegans* treated with *top-2* RNAi bacteria. We first assessed the efficiency of *top-2* knockdown using qPCR. The qPCR results revealed a clear pattern, demonstrating a dose-dependent reduction in *top-2* expression in worms as the concentration of *top-2* RNAi bacteria increased (S1C Fig). We observed a dose-dependent increase in average mobility in worms treated with lower concentrations of *top-2* RNAi bacteria (2.5%, 5%, 10%, Fig. 2D). Conversely, the mobility of worms exhibited a dose-dependent decrease when the concentration of *top-2* RNAi bacteria exceeded 10% (Fig. 2D). Strikingly, the lifespan of worms treated with 5% *top-2* RNAi overlapped with the control, while worms treated with 10% *top-2* RNAi lived shorter than the control (Fig. 2E). Therefore, despite both 5% and 10% *top-2* RNAi treatments exhibiting a beneficial effect on mobility in worms, these did not translate into improvements in lifespan (Fig. 2D–E). To further investigate the role of *top-2* in the lifespan-extending effects of amonafide, we conducted lifespan assays under *top-2* knockdown conditions. Our data indicate that worms treated with *top-2* RNAi alone exhibited a significantly shortened lifespan compared to the control group, confirming that *top-2* is essential for the normal health and longevity of *C. elegans* (S1D Fig). When worms were treated with a combination of *top-2* RNAi and amonafide, we observed a lifespan extension compared to worms treated with *top-2* RNAi alone (S1D Fig). However, this extended lifespan was notably shorter than that observed in control worms treated with amonafide alone. These results suggest that amonafide retains some lifespan-extending effects even in the context of *top-2* RNAi treatment, and if beneficial, rather than harmful, to lifespan. Notably however, we detected residual *top-2* mRNA expression in worms cultured on 100% *top-2* RNAi bacteria, indicating that *top-2* was not fully depleted in our study (S1C Fig). This partial knockdown suggests that residual *top-2* activity may contribute to the observed effects of amonafide, and makes it difficult to ascertain conclusively that amonafide operates fully independently of *top-2*. An experiment with a full knock-out of *top-2* would be required to ascertain if amonafide operates fully independently of *top-2*. Nonetheless, our experimental evidence on healthspan and lifespan is highly suggestive that the impact of amonafide on healthspan may not be exclusively attributed to its inhibition of *top-2* and the benefit of amonafide treatment to lifespan is independent of *top-2*.

It is well-documented that AKT-1 phosphorylates DAF-16, retaining it in the cytoplasm and preventing its entry into the nucleus, thereby inhibiting its transcriptional activity. This inactivation of DAF-16 by AKT-1 results in reduced lifespan and lowered resistance to stress, linking AKT-1 activity to aging processes [35]. Given that our compound screen aimed to mimic *AKT1* inhibition, we next investigated the role of *daf-16/*FOXO in amonafide-induced lifespan extension, as *daf-16/*FOXO is a downstream mediator of lifespan extension from reduced *AKT1* levels [36]. To address this, we turned to *daf-16(mu86)* mutant strain of worms which lack a function *daf-16*/FOXO transcription factor. In comparison to the vehicle, amonafide was still able to significantly increase the mobility of *daf-16(mu86)* worms (Fig. 2F). Furthermore, we also observed an extension in the lifespan of *daf-16(mu86)* worms treated with 50 μM amonafide, although the observed beneficial effect was not as pronounced as in the wild type (Fig. 2G). Taken together, we concluded that the mechanism underlying the longevity effect of amonafide differs in part from *AKT1* inhibition, as it is not fully blocked by *daf-16* inhibition. Additionally, mild *top-2* RNAi, which mimics the effect of *top-2* inhibition, increased mobility but not lifespan. This also implies that pathways other than the canonical targets of amonafide are involved in promoting the longevity effect of amonafide treatment (Fig. 2H).

### The transcriptome altered by amonafide exhibits predicted ages of a younger state and activated defense responses

**Fig. 3:**
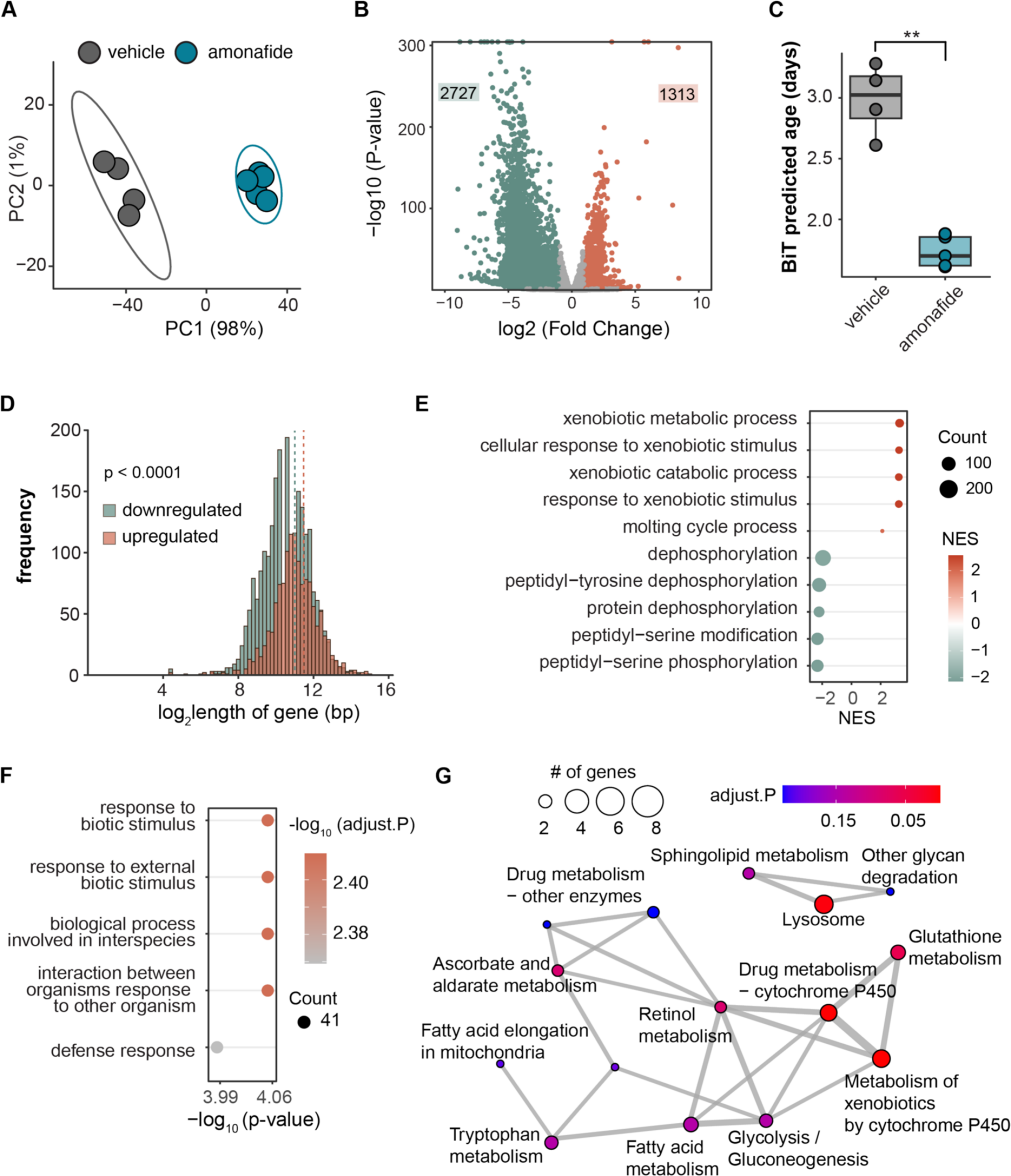
The transcriptome altered by amonafide exhibits predicted ages of a younger state and activated defense responses. **A** PCA plot of the transcriptomic analysis. Samples from amonafide clustered separately from vehicle (DMSO) samples. **B** Volcano plot of genes in amonafide vs. vehicle (adjusted *p*-value < 0.05, absolute log_2_FC > 1, *y*-axis values exceeding 300 were capped at 300). **C** Predicted biological age of worms treated with amonafide or vehicle (DMSO). ** represents a two-tailed *t*-test *p*-value < 0.01. **D** Histogram representing the gene length (log_2_ scale) of DEGs (using adjust *p*-value < 0.05, absolute log fold change > 0.5). Shorter genes are significantly more likely to be downregulated in amonafide-treated worms than longer genes (*p*-value < 0.0001; unpaired *t*-test, dash line represents the median gene length in each group). **E** Top 10 GSEA enriched gene sets in significantly upregulated genes (adjust *p*-value < 0.05, log fold change > 0.5) in amonafide vs vehicle. **F** Top 5 over-representation enriched Biological Process gene sets in significantly upregulated genes (adjust *p*-value < 0.05, log fold change > 0.5) in amonafide vs vehicle. **G** Graph representation of the top enriched KEGG sets in genes upregulated by amonafide

To untangle the mechanism by which amonafide benefits healthspan and lifespan, we conducted RNA sequencing on total RNA isolated from N2 worms treated with or without amonafide. Principal component analysis (PCA) demonstrated that amonafide treatment had a pronounced impact on the transcriptome compared to vehicle (DMSO)-treated worms (Fig. 3A). To further explore the effects resulting from amonafide treatment, we performed differential expression analysis, where we found that compared to the vehicle, 2727 genes were downregulated and 1313 genes were upregulated upon amonafide treatment (adjusted *p*-value < 0.05, Fig. 3B). In line with the longevity effect and the fact that our screen was based on transcriptionally mimicking *AKT1* knockdown, we found that *akt-1* was transcriptionally downregulated following amonafide treatment (S1E Fig).

To better understand how the amonafide-altered transcriptome related to longevity, we explored our RNA-seq data using two recent tools and discoveries that are able to characterize a youthful transcriptome. These included (1) a recently developed binarized transcriptomic aging (BiT age) clock designed to predict the biological age of *C. elegans* based on RNA-seq data [37] and (2) the observation that in global assessment of young and old RNA-seq data, younger transcriptomes possess larger amounts of transcripts from longer genes [38]. We applied the BiT age clock to our RNAseq-dataset, and in line with amonafide’s lifespan extension effects, found a reduction in the predicted biological age of worms treated with amonafide compared to the vehicle (Fig. 3C). Furthermore, assessing the lengths of differentially expressed genes, we observed a significant tendency for longer genes to be upregulated by amonafide, in line with the observation that younger transcriptomes are more likely to express longer genes (Fig. 3D). In summary, the global alterations in the transcriptome aligned with the observed beneficial effects on healthspan and lifespan of amonafide (Fig. 2A, B).

Next, to explore the potential function of genes regulated by amonafide and uncover the mechanism explaining how amonafide improved healthspan and lifespan, we performed Gene Sets Enrichment Analysis (GSEA). We found that the top positively enriched gene sets were “xenobiotic metabolic process,” “cellular response to exobiotic stimulus,” and “xenobiotic catabolic response” (Fig. 3E). Further over-representation analysis on genes upregulated by amonafide revealed top Biological Process gene sets enriched in “response to biotic stimulus,” “biological process involved in interspecies,” and “defense response” (Fig. 3F). Moreover, top KEGG sets enriched in genes upregulated by amonafide included pathways related to “drug metabolism cytostome P450,” “glutathione metabolism,” “retinol metabolism,” and “lysosome,” most of which are involved in responses to biotic stimuli (Fig. 3G). Collectively, these results suggest that amonafide activates key pathways associated with cellular defense mechanisms.

### Amonafide activates gene and protein expression levels in mitochondria-, pathogen-, and xenobiotic-associated responses

**Fig. 4:**
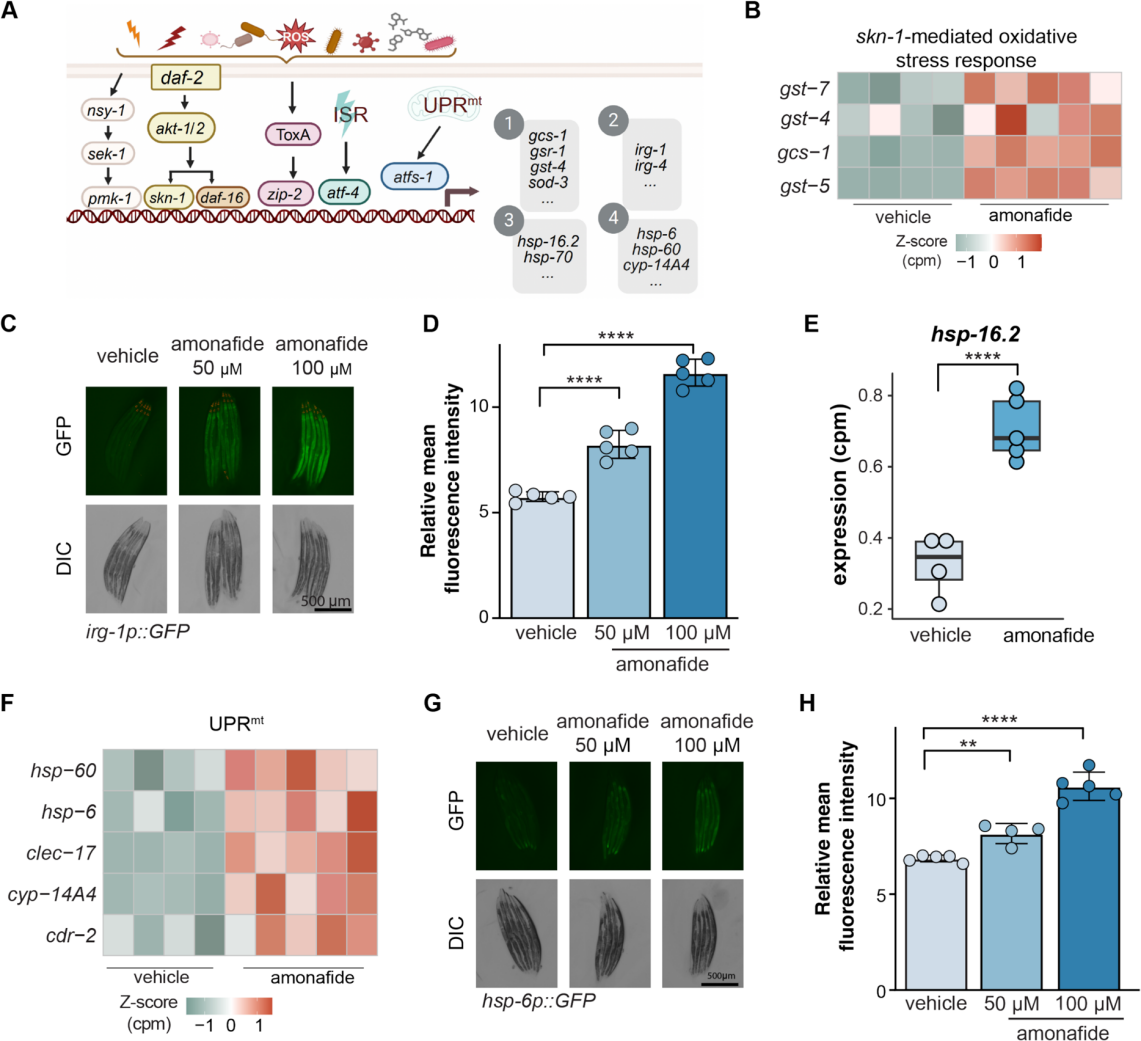
Amonafide activates defense pathways: impact on mRNA and protein levels in pathogen- and xenobiotic-associated responses. **A** Schematic of key longevity-related defense processes in *C. elegans*. The numbered boxes on the right correspond to gene clusters utilized for assessing these defense responses; (1) oxidative stress response, (2) pathogen defense response, (3) integrated stress response, (4) mitochondrial unfolded protein response. **B** Heatmap of key effectors in *skn-1* mediated oxidative stress response in worms treated with vehicle or amonafide. All presented genes were significantly differentially expressed, adjusted *p*-value less than 0.05. **C** Representative fluorescence images of *irg-1p::GFP* reporter worms under vehicle or amonafide (50 μM and 100 μM) on day 1. Scale bar = 500 μm. **D** Quantification of relative fluorescence intensity of *irg-1p::GFP* reporter worms under vehicle or amonafide (50 μM and 100 μM) on day 1. **E** Boxplot of gene expression of *hsp-16.2* under vehicle or amonafide. **** represents a two-tailed *t*-test *p*-value < 0.0001. **F** Heatmap of key effectors of UPR.^mt^ in worms treated with vehicle or amonafide. All presented genes were significantly differentially expressed, adjusted *p*-value less than 0.05. **G** Representative fluorescence images of *hsp-6p::GFP* reporter worms under vehicle or amonafide (50 μM and 100 μM) on day 1. Scale bar = 500 μm. **H** Quantification of relative fluorescence intensity of *hsp-6p::GFP* reporter worms under vehicle or amonafide (50 μM and 100 μM) on day 1. **D**, **H** Each dot represents a biological replicate. The statistical analysis was performed using a two-tailed *t*-test. **** represents *p*-value < 0.0001, *** represents *p*-value < 0.001, and ** represents *p*-value < 0.01

To further dissect the stress responses induced by amonafide treatment, we examined the transcriptional changes in specific key stress response pathways associated with longevity. These pathways include p38 MAPK pathway, *skn-1*-mediated oxidative stress response, *daf-16*-mediated antioxidant activity, *zip-2*-mediated pathogen defense response, *atf-4*-mediated integrated stress response (ISR), and the *atfs-1*-mediated mitochondrial unfolded protein response (UPR^mt^). The p38 MAPK pathway, known for promoting pathogen resistance, contributes to the longevity observed in *daf-2* mutants [39]. *skn-1-*mediated oxidative stress response plays a central role in various genetic and pharmacologic interventions that promote longevity in *C. elegans* [40]. *daf-16* is essential for the longevity induced by *akt-1* inhibition [41]. The *zip-2*-mediated infection defense response is critical for survival following pathogen infection [42]. Meanwhile, *atf-4*, the transcriptional effector of ISR, is required for longevity induced by the global protein synthesis stress [43]. Additionally, *atfs-1*-mediated UPR^mt^ is necessary for lifespan extension induced by mitonuclear protein imbalance and the mitochondrial unfolded protein response [44]. Collectively, these transcription factors and pathways play crucial roles in regulating defense responses, lifespan, and aging in *C. elegans*, and may therefore be integral for lifespan or healthspan extension resulting from amonafide treatment (Fig. 4A).

First, we investigated the expression of p38 MAPK pathway, observing that expression of key components in the p38 MAPK pathway (*pmk-1*, *nsy-1*, and *sek-1*) under amonafide treatment remained comparable between amonafide treatment and the vehicle (S1F Fig). Next, we explored differentially expressed genes related to the *skn-1*-mediated oxidative stress response. Notably, the expression of key effectors of *skn-1*, such as *gcs-1*, *gst-4*, *gst-5*, and *gst-7*, was significantly upregulated following amonafide treatment (Fig. 4B). A similar pattern was also observed in several *daf-16* targeted genes, including *sod-3* and *mtl-1* [45] (S1G Fig). Proceeding to evaluate the expression of the *zip-2*-mediated infection defense response, we turned to examine the infection response gene *irg-1.* This gene serves as a reporter for *zip-2* activity, being activated by *P. aeruginosa* infection and cadmium poisoning in a *zip-2-*dependent manner [42]. The *irg-1::GFP* worms are a commonly used fluorescent reporter strain [42]. We further observed that the expression of *irg-1::GFP* significantly increased in a dose-dependent manner under amonafide treatment (Fig. 4C, D). We then evaluated the chaperone genes including *hsp-70*, *hsp-16.2*, and *hsp-12.3* that are activated through a variety of stress responses including the *atf-4*-mediated ISR [43, 46]. Upon treatment with amonafide, we observed an increase in *hsp-16.2* levels, while the expression of *hsp-70* and *hsp-12.3* decreased (Fig. 4E, S1H Fig). Finally, to assess the UPR^mt^, we investigated the levels of UPR^mt^-related heat shock proteins 6 and 60 (*hsp-6* and *hsp-60*) and other UPR^mt^ targets, which exhibited a significant increase upon amonafide treatment (Fig. 4F). Further evaluation using an *hsp-6::GFP* tagged reporter revealed an activation of UPR^mt^ in a dose-dependent manner following amonafide treatment (Fig. 4G, H). Taken together, amonafide not only activated the mRNA expression of pathways associated with mitochondria-, pathogen-, and xenobiotic-associated defense response but also elevated the protein level of effectors of *zip-2*-mediated pathogen defense and the UPR^mt^.

### *atfs-1* is required for healthspan and lifespan improvement by amonafide

**Fig. 5:**
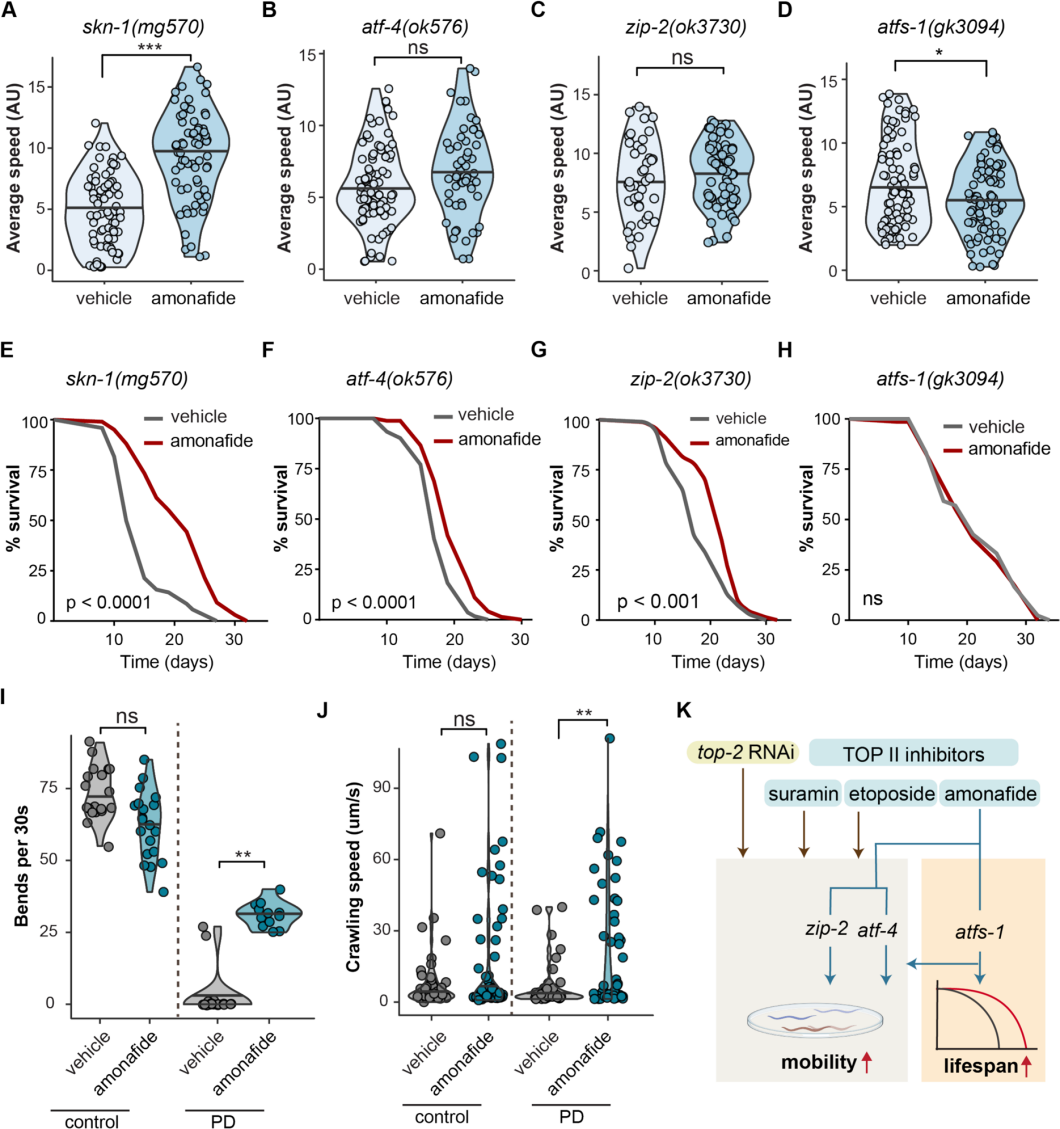
*atfs-1* is required in mediating healthspan improvement and lifespan extension promoted by amonafide. **A**–**D** Violin plot representing the mobility of different defense response deficiency worms including *skn-1(mg570)*, *atf-4(ok576)*, *zip-2(ok3730)*, and *atfs-1(gk3094)* under treatment of vehicle or 50 μM amonafide. *Y*-axis shows the average moving speed of *C. elegans*. The bar in the center of the violin plot represents the median value of mobility. The statistical analysis was performed using a wilcox.test. *** represents *p*-value < 0.001, * represents *p*-value < 0.05, and ns represents not significant. **E** Lifespan curves of *skn-1(mg570)* treated with vehicle or 50 μM amonafide. **F** Lifespan curves of *atf-4(ok576)* treated with vehicle or 50 μM amonafide. **G** Lifespan curves of *zip-2(ok3730)* treated with vehicle or 50 μM amonafide. **H** Lifespan curves of *atfs-1(gk3094)* treated with vehicle or 50 μM amonafide. **E**–**H** *p*-value represents the comparison with the controls calculated using log-rank test. **I**–**J** The violin plot displays the thrashing frequency and crawling speed of control worms (UM9) and PD worms (Parkinson’s disease worm, UM10) under vehicle (DMSO) or amonafide 50-μM treatment. The statistical analysis was performed using a two-tailed *t*-test. ** represents *p*-value < 0.01 and ns represents not significant. **K** The diagram illustrates the study’s findings: TOP II inhibitors and mild RNAi *top-2* enhance mobility, showing promise for promoting longevity. Amonafide exhibits geroprotective effects, relying on *zip-2*, *atf-4*, and *atfs-1* pathways for mobility and *atfs-1* for lifespan

Given the observed activation of defense responses induced by amonafide, we next asked if these were required for the healthspan or lifespan benefits. To do this, we assessed the mobility of worm mutant strains in which defense responses were blocked, with and without amonafide treatment. These included administering amonafide to worms with mutations in *skn-1*, *atf-4*, *atfs-1*, and *zip-2*. Specifically, a dose of 50 μM was used for all mutant lifespan assays. This dose was chosen because it was the lowest dose that demonstrated a lifespan benefit in N2 worms, minimizing potential toxic effects while maximizing the likelihood of observing lifespan extension in mutants. Notably, amonafide exhibited a significant increase in the mobility of *skn-1(mg570)* worms (Fig. 5A). However, the mobility of *zip-2(ok3730)*, *atf-4(ok576)*, and *atfs-1(gk3094)* worms remained similar in both control and amonafide treatment (Fig. 5B–D). This demonstrated that healthspan benefits of amonafide were dependent on *zip-2*, *atf-4*, and *atfs-1.*

We next investigated if lifespan extension following amonafide treatment also depended on these same regulators. Consistent with the mobility assay, amonafide significantly increased the lifespan of *skn-1(mg570)* worms (Fig. 5E). The lifespan of *zip-2(ok3730)* and *atf-4(ok576)* worms also increased upon amonafide treatment, contrary to the findings that the mobility of these worm strains did not change with amonafide treatment (Fig. 5F, G). Noticeably, the lifespan of *atfs-1(gk3094*) worms did not exhibit significant changes with the treatment of amonafide, aligning with our observations in the healthspan assay, where the average mobility of *atfs-1(gk3094)* worms remained similar between amonafide treatment and control conditions (Fig. 5D, H). This demonstrated that lifespan extension resulting from amonafide treatment was dependent on *atfs-1.*

Due to the essential role of the UPR^mt^ activator *atfs-1* in mediating both healthspan and lifespan effects in amonafide treatment, we next aimed to investigate the therapeutic potential of amonafide on a disease model where activation of the UPR^mt^ can play a protective role, namely neurodegeneration [47]. Specifically, we looked at Parkinson’s disease (PD), the second most prevalent age-related neurodegenerative disorder, with aging being the foremost risk factor for the development of idiopathic PD [48]. To evaluate the potential of amonafide in alleviating this age-related disease, we assessed its effects in a PD worm model. Using the UM10 worm model with pathological α-synuclein accumulation [49], we measured thrashing and crawling behavior to represent the disease state. Remarkably, we found that the thrashing frequency and crawling speed demonstrated a statistically significant increase in amonafide-treated PD worms compared to vehicle-treated PD worms (Fig. 5I, J). Taken together, our work suggests a potential therapeutic approach to treating age-related neurodegenerative disorders through treatment with amonafide.

## Discussion

Modulation of IIS components, particularly through reduced AKT-1 activity and activation of DAF-16, promotes stress resistance, metabolic homeostasis, and extended lifespan. Inhibition of *AKT1/akt-1* as a geroprotective intervention extends lifespan in both *C. elegans* and mice [23, 50]. In this study, we used this paradigm to screen for small molecules that may produce similar effects. This approach successfully identified multiple topoisomerase inhibitors with the potential to extend healthspan in *C. elegans*. Among these, the topoisomerase inhibitor amonafide demonstrated the ability to extend both healthspan and lifespan. Inhibition of *top-2* through RNAi was also capable of extending healthspan. Dissecting the mechanism of amonafide—obtained through RNA-seq, fluorescent reporters, and genetic epistasis—revealed that activation of mitochondria-, pathogen-, and xenobiotic-associated defenses played pivotal roles in healthspan and lifespan improved by amonafide. While multiple of these pathways were required for healthspan extension, including *atfs-1*, *zip-2*, and *atf-4*, remarkably, lifespan extension was uncoupled from these and only *atfs-1* was clearly required for amonafide-induced longevity (Fig. 5K). Finally, we demonstrated that amonafide reduced disease symptoms in a PD model in *C. elegans*, specifically related to motility of the worms, suggesting that more studies are warranted to understand if amonafide could be a treatment for PD.

Our investigation on topoisomerase inhibitors began with three distinct compounds, namely suramin, etoposide, and amonafide. All three exhibited a positive impact on healthspan. Additionally, our examination of mild *top-2* RNAi also revealed an increase in healthspan. These findings underscore the potential of topoisomerase II inhibitors as novel categories of healthspan-promoting agents. Given that topoisomerase II inhibitors constitute a diverse category of compounds that interfere with the function of *TOP2*/*top-2* as well as other targets, and exhibit distinct chemical structures and properties [29], it would be worth exploring additional topoisomerase inhibitors for their influence on lifespan and healthspan. Our findings also emphasized the potential of healthspan and lifespan to be uncoupled, in line with other recent findings that locomotion can be uncoupled from lifespan across genetic backgrounds [51]. Dissecting how amonafide—but not other topoisomerase inhibitors—elicit both healthspan and lifespan benefits would therefore also be of future interest. Meanwhile, topoisomerase inhibitors and DNA-intercalating agents can introduce off-target effects, including genotoxicity and the potential to disrupt normal DNA replication and repair processes [52, 53]. Amonafide, in particular, has been associated with dose-dependent myelosuppression in clinical studies [54, 55]. These limitations highlight the need for careful dose optimization and the development of derivatives with improved specificity to reduce adverse effects.

Topoisomerase plays a crucial role in mediating various DNA processes, such as regulating epigenetic modifications, controlling DNA twisting, unwinding, and resolving knots, all essential for the correct functioning of genetic material [56]. An upregulation in the expression of several genes involved in responding to DNA damage and repair has been linked to increased lifespan in model organisms [57]. A recent study highlighted the crucial role of *atfs-1* in balancing genome expression and maintenance in the mitochondria, protecting against the decline in animal behavior caused by mtDNA damage [58]. In line with this, we identified that *atfs-1* is required for the lifespan extension induced by amonafide. Thus, two facets of *atfs-1*’s function may contribute the longevity improved by amonafide: (a) *atfs-1*-mediated UPR^mt^ and (b) *atfs-1*-mediated DNA damage repair responses. Further work to dissect these mechanisms is warranted.

Pathogen- and xenobiotic-associated defenses play pivotal roles in an organism’s adaptive response to stress, encompassing challenges from internal metabolites and external pathogens or xenobiotics [59]. Facing these challenges, the host activates intricate defense mechanisms to counteract potential threats and restore the cellular homeostasis [59]. Our results, obtained through RNA-seq and fluorescent reporters, demonstrated that amonafide activated a diverse array of pathogen- and xenobiotic-associated defense responses. This aligns with the concept of hormesis, where mildly pre-activated stress responses enhance immunity and xenobiotic defenses, promoting host longevity [60–62]. Consistent with this theory, our data suggests that amonafide treatment promotes longevity of the host by stimulating mitochondria-, pathogen-, and xenobiotic-associated defenses. These pre-activated defenses equip the host to respond more efficiently to subsequent challenges, thereby contributing to an overall enhancement of host resilience and longevity.

Amonafide, acting as a DNA intercalating agent disrupting topoisomerase activity, is currently undergoing a phase III clinical trial for secondary acute myeloid leukemia [63]. Despite its recognized anti-cancer effects, there has been no previous association between amonafide and the regulation of longevity, nor with amonafide and treatment of PD. In this study, we present evidence that amonafide possesses pronounced geroprotective properties, complementing its established anti-cancer traits, and demonstrate that amonafide may also serve as treatment for PD, though further experiments are warranted. Since our investigations were conducted in *C. elegans*, future research in higher organism models will be crucial to understand the broader geroprotective effects of amonafide and potential to serve as a treatment for PD. In optimizing towards human translation, special care needs to be given in long-term administration of a DNA intercalating drug such as amonafide, since a side effect—if not properly administered—may be DNA damage and therefore cancer. Interestingly, recent findings have suggested that neurodegenerative diseases may be treated by activation of the UPR^mt^, which relies on *atfs-1*, the mechanism that our study revealed amonafide to operate through [47]. Therefore, considering amonafide as a potential therapy for PD deserves further investigation, with properly designed additional studies to add support.

Certain limitations exist to our study. Firstly, drug treatment was initiated at the L1 stage in *C. elegans*, introducing potential confounding factors that may influence the observed effects on lifespan. Starvation during development, which occurs prior to the introduction of the drug, may impact longevity or interact with the drug to modulate its effects, factors which were not investigated in our work. Likewise, it remains unclear whether the observed lifespan extension results from an interaction of the drug with developmental processes or if it acts directly on aging mechanisms. Future experiments, such as delaying drug treatment until adulthood, will be necessary to distinguish between these possibilities and provide a clearer understanding of the drug’s mode of action. Secondly, in our experiments, 5-FU was included in the agar plates used to culture the worms, which is common practice in *C. elegans* lifespan experiments in order to prevent the outgrowth of progeny. While 5-FU was added in both control and treatment groups to eliminate any potential confounding effects on the comparison, exploring the effects of amonafide in the absence of 5-FU would be a valuable direction for future research. Thirdly, in this study, we focused on screening compounds with transcriptomic signatures resembling AKT-1 inhibition rather than directly testing known AKT-1 inhibitors for their effects on longevity. While this approach allowed us to identify novel candidates such as amonafide, it did not address the potential pro-longevity effects of existing AKT-1 inhibitors. Evaluating known AKT-1 inhibitors represents an important direction for future research. Fourthly, while our study found most affects of amonafide to occur likely independently of topoisomerase II, it would be of interest in future studies to understand biochemically if a physical interaction is occurring with amonafide and topoisomerase II in *C. elegans*. Finally, while our study identified amonafide to provide benefit to motility of the UM10 Parkinson’s worm model, we did not perform further analysis or western blot of pathological α-synuclein accumulation. Therefore further studies are needed to explore the extent to which amonafide provides benefit for Parkinson’s disease.

## Methods

### *C. elegans* strains and maintenance

*C*. *elegans* strains used in this study are as follows: Bristol N2, VC3056 *[zip-2(ok3730)]*, GR2245 *[skn-1(mg570)]*, RB790 *[atf-4(ok576)]*, CF1038 *[daf-16(mu86)]*, VC3201 *[atfs-1(gk3094)]*, AU133 *agIs17 [myo-2p::mCherry* + *irg-1p::GFP]*, SJ4100 *zcIs13 [hsp-6p::GFP* + *lin-15(* +*)]*. These strains were obtained from the *Caenorhabditis* Genetic Center (CGC). Control (UM9): *unkIs11[dat-1p::GFP]*, α-Syn (UM10): *unkIs7 [aex-3P::asyn(A53T), unkls11 [dat-1P::GFP]*. UM9 and UM10 were generously provided by the lab of Prof. Garry Wong, University of Macau [49]. All the *C*. *elegans* strains were maintained at 20 °C on standard nematode growth medium (NGM) plates seeded with *E. coli* OP50 unless otherwise indicated. *E. coli* OP50 is cultured overnight in Luria Broth (LB) medium at 37 °C.

### LINCS database compound screen

The compound screening utilizing the LINCS database was performed as previously described [18]. Briefly, the LINCS online library was accessed via the cloud-based software platform CLUE (https://clue.io/) (September 2017). The *AKT1* knock down (*AKT1*^kd^) transcriptional signature was used as the basis for querying compounds that exhibited similar transcriptional signatures across cell lines. We downloaded a ranked list as.gct files (version 1.3), which included summary scores integrating data from cell lines, with scores ranging from − 100 (indicative of an opposing signature) to 100 (indicative of a mimicking signature). A threshold was applied to this list, whereby compounds with a score greater than 90 were considered to match the *AKT1*^kd^ transcriptional signature. Subsequently, we refined this list by identifying drug classes that were statistically enriched (Fisher’s exact test) among the highest-ranked small molecules, which provided insights into the predominant drug classes within our list relative to the entire dataset.

### Pharmacological treatment of Caenorhabditis elegans

All chemicals utilized in the experiments were obtained from Sigma-Aldrich. Suramin (S2671-100MG) was prepared as a 50-mM stock solution in water. Etoposide (341205-25MG) and amonafide (SML2771-25MG) were dissolved in DMSO as 50-mM stock solutions. These solutions were incorporated into plates immediately before pouring, following the specified concentrations. Unless stated otherwise, worms were subjected to compound treatment starting from the L1 stage. Plates were changed at least once a week to maintain continuous exposure to the compounds.

### RNAi treatment

*Top-2* RNAi clone was obtained from the Ahringer RNAi library [64] and confirmed by sequencing. Unless specifically stated, RNAi bacterial feeding experiments were performed from L1 as described [44]. Briefly, gravid adult worms were synchronized by hypochlorite treatment, then plated on NGMi plates (NGM plates with 2 mM IPTG and 25 mg/mL carbenicillin) with a bacterial lawn of either *E. coli* HT115 (RNAi control strain, containing an empty vector) or *top-2* RNAi bacteria in specified concentration. Bacterial cultures were normalized to optical densities at 600 nm.

### Preparation of UV-killed bacteria

*E. coli OP50* cultures grown overnight were plated onto standard NGM plates supplemented with carbenicillin (25 μg/mL) to inhibit bacterial proliferation. The plates were left to dry overnight at room temperature before the bacterial lawn was exposed to 254-nm UV light using a Stratalinker UV Crosslinker (Model 1800, Stratagene, USA) at 999,900 μJ/cm^2^ for 15 min. To ensure complete bacterial inactivation, a sample of the UV-treated *E. coli OP50* was incubated in LB medium overnight at 37 °C, confirming no growth. Plates containing UV-killed bacteria were stored at 4 °C and utilized within 1 week of preparation.

### Healthspan measurements

Worms were age-synchronized using alkaline hypochlorite treatment and subsequently incubated in M9 buffer overnight to get L1 worms. L1 stage worms were then transferred to NGM plates supplemented with compounds. L4 larval stage was transferred to plates containing compounds and 10 μM 5-fluorouracil (Sigma-Aldrich). The assays were conducted at 20 °C, with the L4 stage designated as day 0 of life. To ensure continuous exposure to the compounds, plates were changed biweekly. For each healthspan study, approximately 50 adult worms were transferred to 3 cm NGM plates without bacteria at specified time points to monitor their moving speed. Worms were stimulated by tapping the plate, and their motion was promptly recorded for 200 cycles at room temperature using a Leica M205 FA fluorescent microscope and Leica DFC 365 FX camera. Leica Application Suite X software was employed for image capture, and the wrMTrck plugin for ImageJ was utilized to determine the average moving speed of each worm. Functional assays, including the tracking of worm movement, were carried out on at least two times, with the presented data reflecting one of the replicates.

### Body size measurements

Body size measurements were performed using day 6 worms. This time point was selected because N2 worms, the strain used in this study, are fully matured, have ceased laying eggs, and have not yet begun to exhibit significant mortality. Images were captured using Leica Application Suite X software for monitoring of moving speed, (healthspan assay), and body size was subsequently analyzed using the wrMTrck plugin for ImageJ to determine the average body size in pixels of each worm. Experiments were conducted at least twice, and the presented data represent one of the replicates.

### Lifespan assay

Lifespan assays were performed as previously described [44]. In brief, worms were grown on plates supplemented with compounds until reaching the L4 stage and subsequently transferred to plates supplemented with compounds as described and 10 μM 5-fluorouracil. For *top-2* RNAi, *top-2* RNAi bacteria were mixed with HT115 to the specified concentrations, then seeded on NGMi plates (with or without 5-fluorouracil). Worms were transferred to fresh plates once a week, and after two transfers, no 5-fluorouracil was added to the plates. Worms were examined every other day by prodding with a platinum wire. All lifespan assays were conducted at least twice, with one of the replicates represented in the shown data. Statistical analyses of lifespan were calculated by Log-rank (Mantel-Cox) tests on Kaplan–Meier curves in GraphPad Prism 9. Detailed statistical analysis for lifespan experiments and their respective replicates is provided in Table S2.

### Crawling and thrashing

After the treatment with 100 μM amonafide, worms were transferred on empty NGM agar plates to assess crawling ability. We recorded their free crawling for 60 s at 20 fps with the WF-NTP platform. The crawling speed is analyzed with the WF-NTP software [65]**.** To assess thrashing behavior, worms were transferred to an empty NGM agar plate flooded with M9, and their thrashing frequency was recorded for 30 s at 20 fps. Subsequently, the thrashing frequency was determined by manually counting 25 randomly selected worms per recording.

### RNA isolation and quantitative RT-PCR

RNA extraction and qRT-PCR were performed as previously described with minor modifications [66]. Total RNA from worms was isolated using TRI reagent (Sigma-Aldrich). One microgram of extracted RNA was reverse transcribed into cDNA using the QuantiTect Reverse Transcription Kit (QIAGEN; Venlo, The Netherlands). qPCR was conducted using the LightCyclerⓇ 480 SYBR Green I Master (Roche; Woerden, The Netherlands) and measured with the LightCyclerⓇ 480 Instrument II (Roche). Relative quantifications were normalized to the reference genes *tba-1* and *F35G12.2*. For each condition, more than three independent samples were prepared. All experiments were performed at least twice. A Student *t*-test was used to compare the differences in gene expression between different conditions. Data visualization was conducted using GraphPad Prism 9.

### Fluorescent microscopy analysis

GFP expression and quantification were carried out as described previously [44]. Briefly, around eighty *hsp-6p::GFP* and *irg-1p::GFP* worms (day 1 adults) were mounted on 2% agarose pads in 10 mM tetramisole (Sigma) and examined using a Leica M205 FA fluorescent microscope. The GFP fluorescence was quantified by ImageJ. Experiments were conducted with worms from three different plates. Each experiment was repeated at least twice.

### RNA sequencing

RNA sequencing was conducted as described [18] with minor modifications. N2 worms were synchronized and subjected to amonafide or DMSO treatment from the L1 stage as previously outlined. L4 animals were harvested by washing three times with M9 buffer and two times with water, followed by snap-freezing in liquid nitrogen. Approximately 2000 worms were collected for each sample. Total RNA was extracted as described above in the “RNA isolation and quantitative RT-PCR” section. Contaminating genomic DNA was eliminated using RNaseFree DNase (QIAGEN). RNA quantification was performed using a NanoDrop 2000 spectrophotometer (Thermo Scientific; Breda, The Netherlands). The qualities of RNA samples were checked with TapeStation (Agilent, CA, USA). Sequencing libraries were constructed using the KAPA mRNA HyperPrep Kit (Roche, Switzerland), and paired-end sequencing was performed on the Illumina NovaSeq platform (Macrogen, Seoul, South Korea).

Reads were subjected to quality control FastQC and trimmed using fastp (version 0.23.2) [67] and aligned to the *C. elegans* genome obtained from Ensembl (wbcel235), using STAR2 (version 2.5.4) [68]. The STAR gene-counts for each alignment were analyzed for differentially expressed genes using the R package DESeq2 (version1.32.0) [69] using a generalized-linear model. Variance Stabilizing Transformation data generated by DESeq2 was used for principal component analyses to explore the primary variation in the data. Count data were normalized to counts per million (CPM) using edgeR (version 3.36.0) [70]. Biological process (BP) overrepresentation analysis and Gene Set Enrichment Analysis (GSEA) were performed using Clusterprofiler (version 4.0.5) [71] and org.Ce.eg.db (version 3.13.0). ggplot2 (version 3.4.2) was used to generate heatmaps and various figures. The code was executed in R version 4.1.1.

### Biological age prediction and gene length analysis

Biological age analysis was performed using the Binarized Transcriptomic Aging (BiT Age) clock prediction model [37]. The BiT Age clock model’s specified parameters were employed to process our RNA-seq dataset, which entailed Count-Per-Million normalized read counts annotated with unique Wormbase IDs. This model provided the biological age of the nematodes in hours. These predicted biological ages were then converted to days for graphical representation. The lengths of genes that were differentially expressed (adjusted *p*-value < 0.05) were retrieved from the Ensembl database (wbcel235). Lengths of upregulated and downregulated genes were compared with an unpaired *t*-test.

### Statistics and reproducibility

All the assays were conducted at least twice independently, and the statistical analysis used in this study is described in the figure legends and/or methods. No statistical method was used to predetermine the sample size. Comparison between more than two groups was assessed by using a one-way ANOVA test. Prism 9 (GraphPad Software) was used for statistical analysis of all lifespans, qRT-PCR, and mobility assay experiments. ****p < 0.0001; ***p < 0.001; **p < 0.01; *p < 0.05; n.s., not significant.

## Supplementary Information

Below is the link to the electronic supplementary material.

Supplementary file1 (DOCX 79.5 KB)

Supplementary file2 (DOCX 487 KB)

## Data Availability

The raw RNA-seq data have been deposited at Gene Expression Omnibus (GEO), and the accession number is GSE254627. This paper does not report original code. Any additional information required to reanalyze the data reported in this paper is available from the lead contact upon request.
