# Supplementary material for: Topoisomerase inhibitor amonafide enhances defense responses to promote longevity in C. elegans
Source: GeroScience. 2025 Mar 14;47(3):5185–204. doi: 10.1007/s11357-025-01599-5 (PMC12181488; doi:10.1007/s11357-025-01599-5)
Supplement: Supplementary file 1 — Supplementary file1 (DOCX 79.5 KB) [file 11357_2025_1599_MOESM1_ESM.docx]

**Table S1 Summary of tested topoisomerase II inhibitor.**

| **Name** | **Structure**  (From DrugBank) | **Known Mechanisms** | **Binding domain** | **Application** |
| --- | --- | --- | --- | --- |
| **Suramin** | **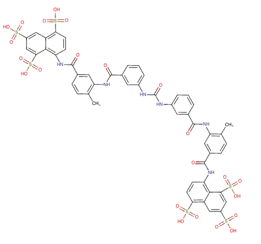** | 1). Inhibit topoisomerase II activity.  2). Antagonist of P2Y purinoceptor 2.  3). Inhibit sirtuin-5  ... | Not characterized (blocks binding of topoisomerase II to DNA) | Treatment of onchocerciasis. |
| **Etoposide** | **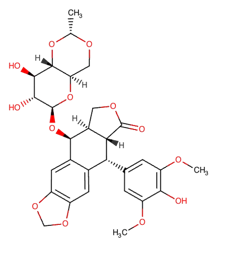** | Inhibits DNA synthesis by forming a complex with topoisomerase II and DNA. | Targets the DNA-binding domain (catalytic core) | 1). Treatment of refractory testicular tumors.  2). First-line treatment in patients with small cell lung cancer. |
| **Amonafide** | **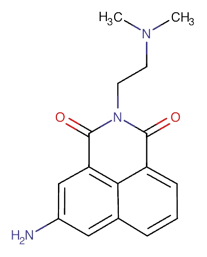** | A DNA intercalating agent disrupting topoisomerase II activity. | Not characterized.  Intercalates with DNA (indirect inhibition of topoisomerase) | 1). Undergoing a phase III clinical trial for Secondary Acute Myeloid Leukemia  2). Under studying in patients with malignant solid tumors. |

**Table S2: Lifespan statistics, related to figure 1, 2, 5 and supplementary figure 1**

| ***C. elegans* strain** | **Treatment** | **Median lifespan (days)** | **Number of animals (died/total)** | **P – value versus control group** |
| --- | --- | --- | --- | --- |
| **Fig 1F** | | | | |
| Wild Type (N2)^*^ | Water vehicle | 18 | 78/100 | ns |
|  | 10 μM suramin | 18 | 77/100 |  |
| Wild Type (N2) | Water vehicle | 18 | 78/98 | ns |
|  | 10 μM suramin | 18 | 74/97 |  |
| **Fig 1G** | | | | |
| Wild Type (N2)^*^ | DMSO vehicle | 19 | 71/100 | ns |
|  | 100 μM etoposide | 19 | 66/100 |  |
| Wild Type (N2) | DMSO vehicle | 19 | 65/100 | ns |
|  | 100 μM etoposide | 19 | 69/100 |  |
| **Fig 1H** | | | | |
| Wild Type (N2)^*^ | DMSO vehicle | 19 | 71/109 | 0.0001 |
|  | 100 μM amonafide | 26 | 87/121 |  |
| Wild Type (N2) | DMSO vehicle | 19 | 77/100 | 0.0001 |
|  | 100 μM amonafide | 24 | 65/100 |  |
| **Fig 2B** | | | | |
| Wild Type (N2) ^*^ | DMSO vehicle | 19 | 140/177 |  |
|  | 50 μM amonafide | 24 | 101/147 | 0.0001 |
|  | 100 μM amonafide | 24 | 126/176 | 0.0001 |
|  | 200 μM amonafide | 24 | 88/130 | 0.0001 |
| Wild Type (N2) | DMSO vehicle | 20 | 73/100 |  |
|  | 50 μM amonafide | 25 | 62/100 | 0.0001 |
|  | 100 μM amonafide | 25 | 57/100 | 0.0001 |
|  | 200 μM amonafide | 22 | 58/100 | 0.0001 |
| **Fig 2C** | | | | |
| Worms fed with UV-killed OP50 bacteria | | | | |
| Wild Type (N2) | DMSO vehicle | 21 | 96/123 |  |
|  | 100 μM amonafide | 26 | 81/118 | 0.0001 |
| Wild Type (N2) | DMSO vehicle | 24 | 95/130 |  |
|  | 100 μM amonafide | 29 | 74/118 | 0.0001 |
| **Fig 2E** | | | | |
| Wild Type (N2)^*^ | HT115 | 19 | 57/100 |  |
|  | 5% *top-2* RNAi | 19 | 67/100 | ns |
|  | 10% *top-2* RNAi | 17 | 44/100 | 0.05 |
| Wild Type (N2) | HT115 | 19 | 59/100 |  |
|  | 5% *top-2* RNAi | 19 | 70/100 | 0.05 |
|  | 10% *top-2* RNAi | 16 | 59/100 | 0.001 |
| **Fig 2G** | | | | |
| *daf-16(mu86)*^*^ | DMSO vehicle | 15 | 58/80 | 0.05 |
|  | 50 μM amonafide | 15 | 58/80 |  |
| *daf-16(mu86)* | DMSO vehicle | 16 | 58/80 | 0.001 |
|  | 50 μM amonafide | 16 | 55/80 |  |
| **Fig 5E** | | | | |
| *skn-1(mg570)*^*^ | DMSO vehicle | 12 | 73/100 | 0.0001 |
|  | 50 μM amonafide | 22 | 67/100 |  |
| *skn-1(mg570)* | DMSO vehicle | 14 | 70/100 | 0.0001 |
|  | 50 μM amonafide | 19 | 70/100 |  |
| **Fig 5F** | | | | |
| *atf-4(ok576)*^*^ | DMSO vehicle | 17 | 59/100 | 0.001 |
|  | 50 μM amonafide | 19 | 75/100 |  |
| *atf-4(ok576)* | DMSO vehicle | 18 | 59/80 | 0.0001 |
|  | 50 μM amonafide | 23 | 54/80 |  |
| **Fig 5G** | | | | |
| *zip-2(ok3730)*^*^ | DMSO vehicle | 17 | 78/100 | 0.001 |
|  | 50 μM amonafide | 22 | 72/100 |  |
| *zip-2(ok3730)* | DMSO vehicle | 16 | 72/100 | 0.05 |
|  | 50 μM amonafide | 21 | 62/100 |  |
| **Fig 5H** | | | | |
| *atfs-1(gk3094)*^*^ | DMSO vehicle | 21 | 44/80 | ns |
|  | 50 μM amonafide | 21 | 37/80 |  |
| *atfs-1(gk3094)* | DMSO vehicle | 22 | 51/100 | ns |
|  | 50 μM amonafide | 22 | 37/100 |  |
| **Control for lifespans in Fig 5** | | | | |
| Wild Type (N2) | DMSO vehicle | 22 | 92/121 |  |
|  | 50 μM amonafide | 27 | 91/114 | 0.0001 |
| Wild Type (N2) | DMSO vehicle | 21 | 83/106 |  |
|  | 50 μM amonafide | 24 | 69/100 | 0.0001 |
| **S1D Fig** | | | | |
| Wild Type (N2) | HT115 | 19 | 62/100 |  |
|  | 100% *top-2* RNAi | 14 | 40/123 |  |
|  | 100 μM amonafide | 21 | 67/121 |  |
|  | 100% *top-2* RNAi + 100 μM amonafide | 19 | 67/110 | 0.0001 |
| Wild Type (N2) ^*^ | HT115 | 19 | 61/100 |  |
|  | 100% *top-2* RNAi | 14 | 37/115 |  |
|  | 100 μM amonafide | 22 | 65/118 |  |
|  | 100% *top-2* RNAi + 100 μM amonafide | 19 | 54/115 | 0.0001 |

*Experiment represented in figure.
