# Supplementary material for: Topoisomerase inhibitor amonafide enhances defense responses to promote longevity in C. elegans
Source: GeroScience. 2025 Mar 14;47(3):5185–204. doi: 10.1007/s11357-025-01599-5 (PMC12181488; doi:10.1007/s11357-025-01599-5)
Supplement: Supplementary file 2 — Supplementary file2 (DOCX 487 KB) [file 11357_2025_1599_MOESM2_ESM.docx]

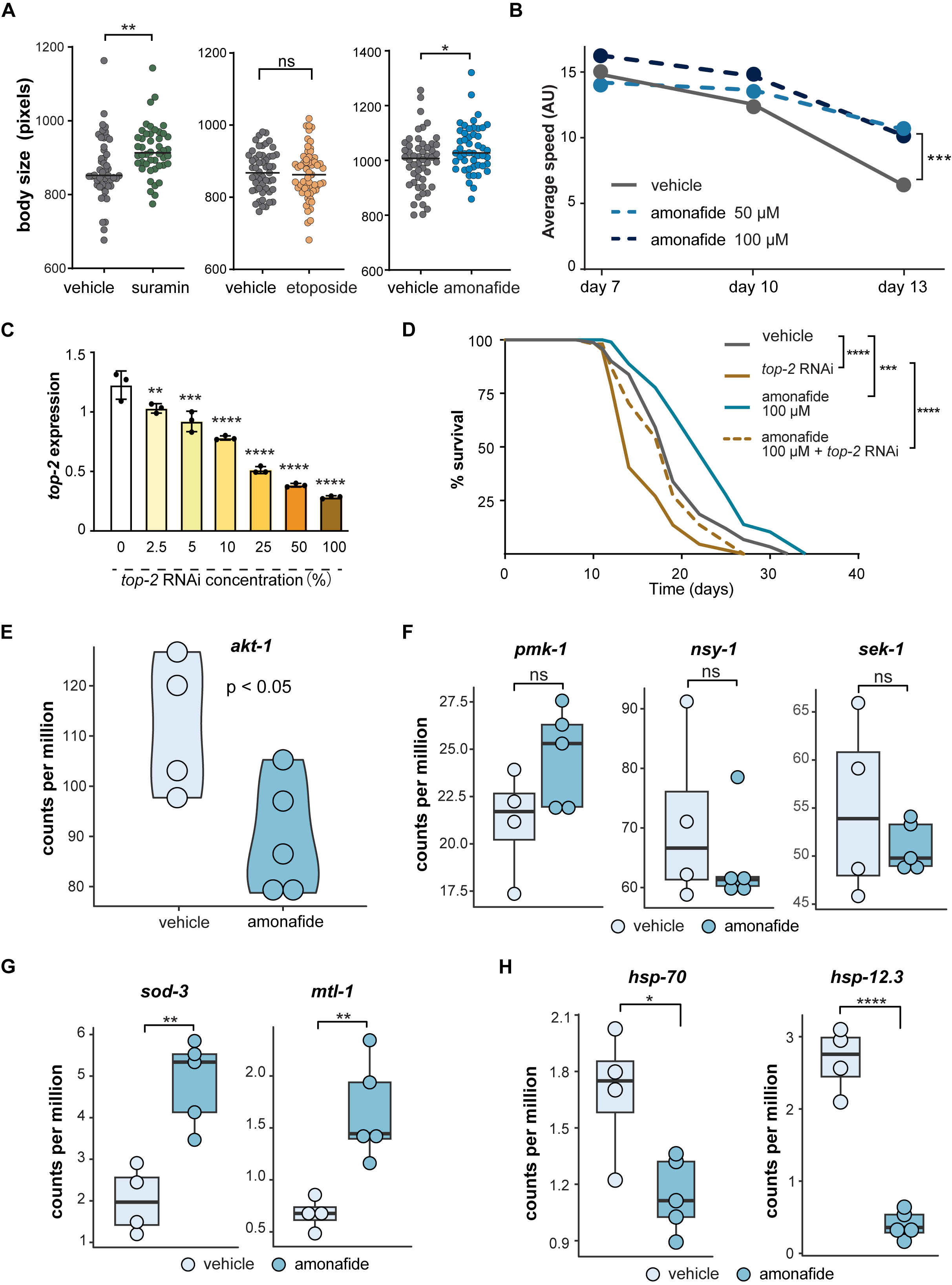


**Supplementary fig 1. Effect of amonafide on gene expression, mobility and lifespan.** (A) Body size of worms treated with 100 μM suramin, 100μM etoposide, 100 μM amonafide and DMSO as control. Each data point represents the body size of an individual worm, measured on day 6. Statistical analysis was conducted using a two-tailed t-test. ** indicates p < 0.01, and * indicates p < 0.05. (B) Line chart illustrating the mobility trend in worms treated with amonafide and vehicle. Statistical analysis at day 13 was performed using a Wilcoxon test. *** represents p-value < 0.001. (C) mRNA expression (measured by qPCR) of *top-2* in N2 worms. X-axis represents the proportion of *top-2* RNAi bacteria, which was supplemented with control HT115 bacteria to reach 100% and provide equal amounts of bacterial food to the worms. The statistical analysis was performed using a One-Way ANOVA test followed by Tukey post hoc test; groups were compared to vehicle. **** represents p-value < 0.0001, *** represents p-value < 0.001, *** represents p-value < 0.01. (D) Lifespan curves of *C. elegans* (N2) fed with control HT115 bacteria or 100% *top-2* RNAi bacteria, with or without supplementation of 100 μM amonafide. P-values were calculated using the log-rank test for comparisons with the control group. *** represents p-value < 0.001, **** represents p-value < 0.0001. (E) mRNA expression of *akt-1* under treatment with DMSO and amonafide. A two-tailed t-test p-value < 0.05. (F) Boxplots presenting mRNA expression of key genes in the p38 MAPK pathway (*pmk-1*, *nsy-1*, and *sek-1*) under DMSO and amonafide treatment. "ns" indicates a two-tailed t-test not significant. (G) Boxplots showing mRNA expression of *daf-16* effectors (*sod-3* and *mlt-1*) under DMSO and amonafide treatment. ** represents a two-tailed t-test p-value < 0.01. (H) Boxplots showing mRNA expression of *hsp-70* and *hsp-12.3* under DMSO and amonafide treatment. ** represents a two-tailed t-test p-value < 0.01.
